# Salt content of sauces in the UK and China: cross-sectional surveys

**DOI:** 10.1136/bmjopen-2018-025623

**Published:** 2019-09-23

**Authors:** Monique Tan, Feng J He, Jingmin Ding, Yuan Li, Puhong Zhang, Graham A MacGregor

**Affiliations:** 1 Wolfson Institute of Preventive Medicine, Barts and The London School of Medicine & Dentistry, Queen Mary University of London, London, UK; 2 Nutrition and Lifestyle Program, The George Institute for Global Health at Peking University Health Science Center, Beijing, China; 3 Faculty of Medicine, University of New South Wales, Sydney, New South Wales, Australia

**Keywords:** salt reduction, reformulation, sauces, salt targets, UK, China

## Abstract

**Objectives:**

(1) To assess the changes in the salt content of sauces in the UK in the past 10 years; (2) to compare the salt content of sauces in China with equivalent products sold in the UK and (3) to calculate the proportion of sauce products meeting the salt targets set by the UK Department of Health (DoH).

**Design:**

Cross-sectional surveys from the nutrition information panels of sauces.

**Setting:**

Major retailers in London, Beijing and Shijiazhuang operating at data collection times.

**Main outcome measure:**

Salt content of sauces.

**Results:**

Relative change in the median salt content of UK products ranged from −70.6% to +3.0% in sauces for which salt targets were set, whereas it ranged from −27.1% to +111.5% in sauces without targets. Median salt contents were on average 4.4-fold greater in Chinese sauces compared with their UK equivalents surveyed during the same period (2015–2017). Only 13.4% of the Chinese products met the UK 2017 salt targets, compared with 70.0% of UK products.

**Conclusion:**

In the UK, the target-based approach contributed to the reduction in the salt content of sauces over the course of the past 10 years. Currently, large variations in salt content exist within the same categories of sauces and 70% of the products have met DoH’s 2017 targets, demonstrating that further reductions are possible and lower salt targets should be set. In China, salt content of sauces is extremely high with similarly large variations within same categories of sauces, demonstrating the feasibility of reducing their salt content. As processed foods (including sauces) are expected to become an important contributor to salt intake in China, national salt reduction efforts such as setting salt targets would be a valuable, proactive strategy.

**Strengths and limitations of this study**

- In the UK, the salt content of sauces declined over time when salt targets were set—an achievement that reinforces the evidence supporting an incremental and target-based approach to salt reduction.
- In China, sauces contained much more salt than their UK equivalents and with the rising consumption of processed foods, there is an urgent need to set salt targets following the UK model.
- This is the first study to compare food products in China and the UK at category level with data collected following the same methodology and over the same years.
- Comparing the sauces at the more precise level of categories (eg, ‘light soy sauces’, ‘dark soy sauces’) instead of food groups (eg, ‘sauces’) provided more meaningful results, essential for any reformulation effort from the food industry.
- Since the data were collected from nutrition information panels, the main limitation of this study is the reliance on their presence (14.9% of the Chinese products did not have a nutrition label) and their accuracy.

## Introduction

There is much evidence that dietary salt intake is the major cause of raised blood pressure,^1^ which, in turn, increases the risk of cardiovascular disease, the leading cause of death and disability in the world.^2^ The WHO has set a global target of 30% reduction in salt intake by 2025, and recommends that adults consume less than 5 g of salt per day.^3^

The UK has pioneered a successful salt reduction programme predominantly by setting incremental salt targets for over 85 categories of food. The targets are voluntary and their enforcement is closely monitored by governmental and non-governmental organisations. Studies have shown that this target-based approach has led to a reduction in the salt content of many food products by 20%–50% over a period of 10 years.^4–6^ As a result, the mean salt intake in the population has fallen by 15% from 9.5 g/d in 2003 to 8.1 g/d in 2011, which was accompanied by a significant decrease in population blood pressure and cardiovascular mortality.^7^

In China, salt intake is very high with an average of 12–14 g/d. While most of the salt in the Chinese diet is added by the consumers during cooking (making up 70%–75% of the total salt intake^8 9^) or consumed in the form of sauces, for example, soy sauce (making up 6%–16% of total salt intake^8–10^), there has been a rapid increase in the consumption of processed food, particularly in urban areas.^9 11 12^ To reduce population salt intake in China, it is vital to encourage individuals to reduce the amount of salt used during food preparations at home; at the same time, a reduction in the amount of salt added to processed foods by the industry will also play an important role. To our knowledge, no specific salt reduction target has been set for any food categories in China.

So far, two studies have shown that Chinese food products contained more salt than their counterparts in the UK. One focused on instant noodles and found that out of 10 countries, those from China contained most salt.^13^ The other compared 11 food groups and found that eight of them contained more salt in China than in the UK: one of the largest gaps was in the ‘sauces and spreads’ food group, where Chinese products had 4.4 times the amount of salt of UK ones. This comparison was, however, made for the entire food group of ‘sauces and spreads’, thus making it impossible to determine more specifically which condiments were the most responsible for this large difference.^14^

Given this discrepancy and the substantial contribution of sauces to salt intake in China, this study aimed to provide a more detailed comparison between UK and Chinese sauces. The first objective was to assess the change in the salt content of sauces in the UK in the past 10 years; the second was to compare the salt content of sauces in China with equivalent products in the UK; and the third was to calculate the percentage of products that would meet the voluntary 2017 salt targets set by the UK Department of Health (DoH).

## Methods

### Data collection

Data were collected from grocery retail stores in Beijing and Shijiazhuang between 2015 and 2017 (Beiguo, Tiankelong, Xinyulou, Wal-Mart, Carrefour, Youghui Superstores) and London between 2008 and 2018 (Aldi, ASDA, Lidl, Marks and Spencer, Morrisons, Sainsbury’s, Tesco, The Co-operative, Waitrose). Salt content values were obtained from the nutrition information panels and reported in gram per 100 gram or millilitre of food (g/100 g or mL).

In this study, any liquid, paste, powder or semiliquid mixture added during food preparation or at the table to provide a savoury flavour were considered as ‘sauces’. All sauce categories that could be found both in China and the UK were included. Products were excluded if (i) the value for salt or sodium content was missing, (ii) they were duplicates or identical products of different package sizes (in such cases, only one product was kept per year) or (iii) sample size was <5 when stratified by category and year. Spreads were excluded since they are not consumed in comparable ways between the two countries.

This resulted in the inclusion of 18 categories, which are described in online supplementary table 1, along with their corresponding UK salt targets.

10.1136/bmjopen-2018-025623.supp1 Supplementary file 1

### Data analysis

All sodium values were converted to salt, using the conversion factor 1 g sodium (Na)=2.5 g salt (NaCl).

All analyses were carried out at category level instead of food group level (eg, ‘ketchups’ was considered as a category, and ‘sauces’ as a food group).

#### Descriptive statistics

Mean, SD, median, IQR and range of salt content in g/100 g or mL were calculated for both countries, for each sauce category, and each year. The total number and percentage of products meeting the UK DoH’s maximum salt targets for 2010, 2012 and 2017 were also calculated.

#### Inferential statistics

Since normal and symmetric distribution could not be assumed due to the small sample sizes, all analyses were conducted using median values and with the appropriate non-parametric tests. To assess the influence of reduced-salt versions of products, all analyses were conducted with and without them.

Within-country analyses were conducted for the UK for the years 2008–2018. For the categories with equality of variance (assessed with Fligner-Kileen tests), the salt content of products was compared between each year with Kruskal-Wallis H tests. When variance was not homogeneous, Mood’s median tests were used. In all cases, post-hoc tests were carried out using Conover-Iman tests with Bonferonni correction. The percentage reduction in median salt content between the oldest salt value recorded and the newest was calculated for each category. No within-country analyses were performed for China since the surveys were carried out within a short time frame (2015–2017) and no change in the products’ salt content was expected to be seen during this period.

Between-country analyses were conducted with Mood’s median tests for the most recent survey (2015–2017). For these analyses, if the same products have been surveyed several times between 2015 and 2017, only the most recent values were kept to avoid over-representation.

A p value of <0.05 was considered significant. All analyses were conducted using R V.3.5.0. Ethics committee approval was not required for the survey of food products.

### Patient and public involvement

This study did not involve any patient or public.

## Results

**Figure 1:**
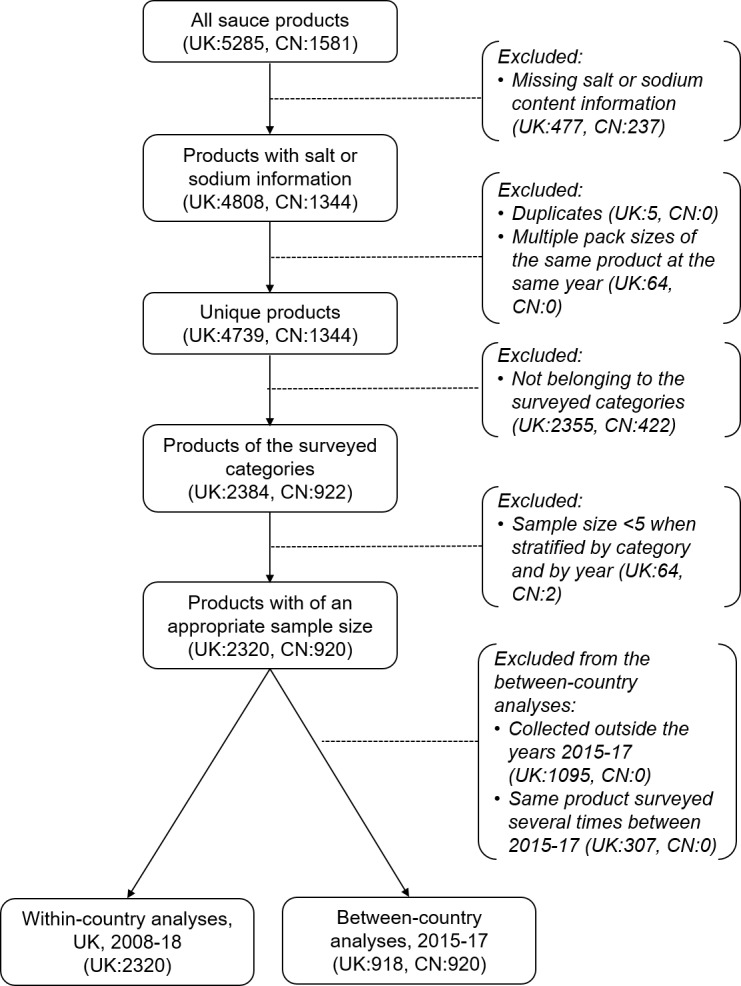
Flow diagram of product selection. UK, United Kingdom; CN, China.

A total of 2320 products were eligible for inclusion in the within-country analyses and 1838 in the between-country analyses (figure 1).

In the UK, the two largest categories were pasta sauces (n=794) and curry cooking sauces (n=479). In China, they were bean pastes (n=178) and vinegars (n=170).

### Within-country analyses

Online supplementary table 2 presents the absolute salt content of sauces per country, per category and per year (2008–2018), as well as the change in median salt content of UK products over the years.

In the UK, a general pattern observed in most categories is a general decline in the median salt content between 2008 and 2018, with an interruption between 2013 and 2015 where salt levels either plateaued or even increased (online supplementary figure 1). The change in median salt content over the years reached statistical significance for eight out of seventeen categories (table 1). Comparing the oldest and newest salt value recorded between 2008 and 2018, the median salt content declined in 10 out of the 11 categories for which targets were set. In these categories, the percentage change ranged from a reduction of 70.6% in bean pastes (falling from 3.40 g/100 g in 2008 to 1.00 g/100 g in 2018) to an increase in 3.0% in curry pastes (rising from 3.30 g/100 g in 2010 to 3.40 g/100 g in 2017). On the other hand, such a pattern was not seen in sauce categories without targets, where median salt content increased in four out of six categories. In sauce categories without targets, the percentage change ranged from a reduction of 27.1% in chilli sauces (falling from 3.50 g/100 g in 2008 to 2.55 g/100 g in 2018) to an increase in 111.5% in marinades (rising from 1.13 g/100 g in 2013 to 2.39 g/100 g in 2018). Changes in the salt content of UK oyster sauces could not be examined since their salt content had only been recorded once (in 2017).

**Table 1:** Absolute and relative changes in the median salt content (g/100 g or mL) of sauces in the UK over the period of the past 10 years, per category

Sauce category	Oldest survey			Newest survey			Change from oldest to newest survey
	Survey year	N	Median (g salt per 100 g or mL) (Range)	Survey year	N	Median (g salt per 100 g or mL) (Range)	
Categories with targets							
Bean pastes	2008	5	3.40 (1.70–3.80)	2018	15	1.00 (0.50–4.30)	−70.6%**
Ketchups	2008	14	2.65 (1.08–3.50)	2017	24	1.43 (0.20–3.09)	−46.0%***
Mayonnaises	2008	10	1.50 (1.30–2.05)	2018	12	1.03 (0.78–1.16)	−31.3%***
Salad creams	2008	15	2.10 (0.80–3.10)	2018	7	1.45 (1.20–1.63)	−31.0%
Low-fat mayonnaises	2008	11	2.30 (1.80–2.80)	2018	9	1.60 (0.95–1.69)	−30.4%****
Barbecue sauces	2010	10	1.23 (0.80–2.50)	2018	5	0.88 (0.70–3.40)	−28.5%
Salad dressings	2008	13	1.67 (0.25–3.00)	2018	12	1.29 (0.98–1.60)	−22.8%
Curry cooking sauces	2010	186	0.80 (0.10–4.00)	2018	17	0.75 (0.60–1.60)	−6.3%**
Hoisin sauces	2017	6	1.45 (0.65–8.00)	2018	18	1.40 (0.65–8.00)	−3.1%
Pasta sauces	2009	200	0.80 (0.10–3.00)	2018	7	0.78 (0.50–1.30)	−2.5%*
Curry pastes	2010	27	3.30 (0.97–5.65)	2017	22	3.40 (1.02–14.20)	+3.0%**
Categories without targets							
Chilli sauces	2008	7	3.50 (2.50–4.10)	2018	17	2.55 (0.49–6.30)	−27.1%*
Light soy sauces	2008	19	15.50 (9.00–22.50)	2018	13	13.00 (9.10–18.70)	−16.1%
Dark soy sauces	2008	5	14.25 (14.00–17.20)	2018	9	14.70 (10.51–19.30)	+3.2%
Stocks (as sold)	2013	5	15.00 (14.00–16.65)	2017	11	18.93 (12.16–44.60)	+26.2%
Marinades	2013	7	1.13 (0.28–2.00)	2018	6	2.39 (0.28–11.50)	+111.5%
Vinegars	2013	11	0.00 (0.00–0.10)	2017	4	0.07 (0.00–0.11)	N/A

The percentage change for vinegars could not be computed (divide by zero error). Only sauce categories with five or more products per year are shown.

*p<0.05; **p< 0.01; ***p< 0.001; ****p<0.0001.

### Between-country analyses

In the latest survey (2015–2017), the median salt content was 0.90 g/100 g or mL for all UK sauces and 8.78 g/100 g or mL for all Chinese sauces. In both countries, the lowest salt content was found in vinegars (0 g/100 mL in both UK and China) and the highest in stocks (as sold) (44.60 g/100 g in the UK, 60.25 g/100 g in China).

**Figure 2:**
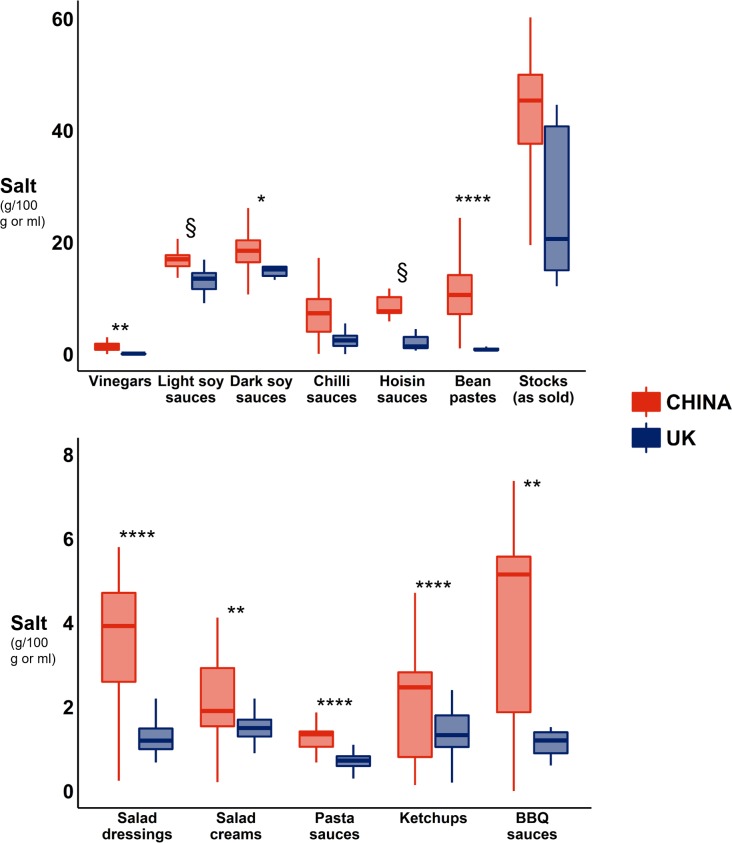
Comparison of the median salt content of sauces between the UK (blue) and China (red), per category, for 2015–2017 (grouped years). Only sauce categories with five or more products in each country are shown. The bottom and top edge of the boxes represent the first and third quartiles; the lines within the boxes represent the medians; and the ends of the bottom and top whiskers represent the minimum and maximum values. §p=0.05; *p<0.05; **p<0.01; ***p<0.001; ****p<0.0001.

The median salt content of sauces was consistently higher in China compared with the UK, across all categories (figure 2). The tests reached statistical significance for all categories except for chilli sauces and stocks (as sold), and was borderline significant (p=0.05) for light soy sauces and Hoisin sauces. Chinese products contained on average 4.4 times the amount of salt of the UK products. The categories with the largest difference in salt content were the vinegars (13.4-fold greater salt content in China), bean pastes (13.1-fold) and oyster sauces (7.2-fold), whereas the categories with the most similar salt content between UK and China were the dark soy sauces (1.2-fold greater salt content in China), light soy sauces (1.3-fold) and mayonnaises (1.3-fold). Online supplementary table 3 presents the salt content of the sauces per country and category, for the years 2015–2017, as well as the proportion of those products meeting the UK DoH’s maximum 2017, 2012 and 2010 salt targets.

While most UK products (70.0%) met the UK 2017 maximum salt targets, only 13.4% of the Chinese products did. The sauce categories that were the farthest from meeting the UK 2017 targets in China were the mayonnaises and curry pastes (where 0% of the products met the target), followed by hoisin sauces (4.5%). It is worth noting that even in the Chinese categories that complied best with the UK 2017 target, only a minority of the product would have met the target: 34% of the salad creams, 31.8% of the ketchups and 18.2% of the barbecue sauces. Even when compared against older UK maximum salt targets (2010 and 2012), less than half of the Chinese products would have complied (15.1% and 44.1% across all categories, respectively) (figure 2, online supplementary table 3).

There were 17 reduced-salt products (seven light soy sauces, two stocks, eight ketchups), only found in the UK; no such product was sold in China. Excluding them from the analyses did not affect the results, except for light soy sauces in the between-country analyses: once the reduced-salt versions removed, the difference in median salt content became smaller and statistically non-significant (online supplementary table 3).

## Discussion

Our study produced several important findings: (i) a large decline in the median salt content of UK sauces over the course of the past 10 years has been observed in most categories for which salt targets were set; (ii) according to the latest survey, sauces in China consistently contained more salt compared with their UK equivalents, across all categories surveyed and (iii) while only 13.4% of the Chinese products would meet the UK 2017 salt targets, 70.0% of the UK sauces did.

The findings of our within-country analyses concur with the large salt declines previously found in many food categories in the UK since 2001,^4^ two examples being the 20% reduction in breads between 2001 and 2011^5^ and the 47% reduction in breakfast cereals between 2004 and 2015.^6^ ^4^ However, since those previous investigations aimed at monitoring the progress of the UK salt reduction strategy, they solely focused on food categories for which salt targets were set—thus failing to capture the change in the salt content of foods without targets. In contrast, the present study also surveyed products for which no target has been set and found that their salt content increased in most categories (relative change ranging from −27.1% to +111.5%) over the course of the past 10 years, while it mostly decreased (relative change ranging from −70.6% to +3.0%) in the categories with targets. This finding reinforces the evidence that salt targets are effective. It is also worth noting that in the survey of breakfast cereals,^4^ the rate of the salt decline flattened between 2012 and 2015; this matches with the interrupted decline observed in the present study between 2013 and 2015. This interruption could be a knock-on effect of the Public Health Responsibility Deal, launched in 2011, where the responsibility for improving nutrition was moved from the Food Standards Agency (an independent agency) to the food industries.^15^

The results of our between-country analyses are also concordant with those of previous studies, where products sold in China usually contained more salt than those found on the UK market.^13 14^ In Huang *et al*’s food group-level comparison, 77% of the products they included in the food group ‘Sauces and spreads’ were cooking sauces, gravies and stocks in China (categories known to have higher salt content than other sauces), compared with 55% in the UK.^14^ This ‘product mix’ limits the usefulness of their results. The present study not only addressed this bias but also went a step further by also ensuring comparability within the categories, for example, by performing analyses with and without reduced-salt products, and by excluding powder products in predominantly liquid categories (eg, in chilli sauces) and vice-versa (eg, in stocks). Additionally, our findings also align with those of Farrand *et al*, who observed that a higher proportion of products met salt targets in countries where targets were set in comparison to countries without targets.^13^

On the whole, all our findings consistently suggest that salt targets (even voluntary ones) can be an effective policy tool as long as there are clear mechanisms for monitoring and penalties for non-compliance: first, the largest declines in salt content over the period of the past 10 years were seen in sauce categories for which targets were set; second, over the same period of time, salt content increased in most categories without targets; third, sauces in the UK systematically contained less salt than their sauces in China. The third point clearly demonstrates the technical feasibility of reducing the salt content of sauces in China via product reformulation. As to consumers’ acceptance, the evidence suggests that a gradual reduction in salt content is not detectable: for instance, in a randomised controlled trial, a progressive 25% salt reduction in bread over 6 weeks went largely unnoticed by the participants.^16^ Moreover, consumers’ taste preference alone cannot explain the large cross-national discrepancies since there were also vast differences in salt content within the same sauce categories in China: for example, salt content ranged from 1.0 to 37.5 g/100 g in Chinese bean pastes, and from 0.1 to 25 g/100 g in Chinese chilli sauces.

This is the first study to compare food products in China and the UK at category level with data collected following the same methodology and over the same years. The more refined classification of the products into categories instead of food groups allowed for more meaningful comparisons by avoiding the issue of ‘product mix’. The main limitations of this study are its reliance on the presence of nutrition information panels (14.9% (n=237) of the Chinese products did not have any nutrition label) as well as their accuracy. Another limitation is that our databases did not contain sales information, preventing us from identifying the most purchased products and from using the UK sales-weighted average salt targets. It is also important to note that the relative changes in median salt content over the course of the past 10 years could also be explained by the introduction of new, reduced-salt sauces in the market. However, excluding reduced-salt products from our analyses did not change our within-country results.

This study has two main implications: first, lower salt targets are needed in the UK since 70% of the sauces surveyed in the present study already met the 2017 targets—the large variations in the salt content within UK sauce categories clearly indicating that further salt reduction is entirely possible; second, there is an urgent need to set salt targets in China, with the possibility to adopt the UK model. The UK’s target-based approach ensures a level playing field for the food industry (ie, all companies working towards the same targets) and follows a policy of unobtrusive reformulation, which resulted in a reduction in population salt intake while consumers continue to eat the same foods—a clear advantage over launching new reduced-salt products for example. Should this model be adopted in China and the amount of salt be reduced incrementally by 20% every 2–3 years, it would take on average 11–17 years for Chinese sauces to reach the current median salt levels of UK sauces (ranging from 2 to 3 years for dark soy sauces to 24–36 years for bean pastes and vinegars). Future research should include an updated measure of the contribution of different foods to the salt intake of the Chinese population. In the meantime, any reduction in the salt content of Chinese foods would be beneficial. Additional studies comparing the salt content of major contributors to salt intake in China to benchmarks such as the UK products would be helpful in highlighting where reformulation can be done, both technologically and from the consumers’ perspective.

In conclusion, the salt content of sauces in the UK has declined over the past decade, which is an achievement that reinforces the evidence supporting an incremental and target-based approach to salt reduction. This strategy must be sustained in the UK and started in China, where sauces contained on average 4.4 times the amount of salt of their UK equivalents. Since sauces are top contributors to salt intake, even relatively small reductions in their salt content would be impactful. Failing to reduce the salt content of sauces in China would be detrimental to the Chinese population as well as to the population of the countries where Chinese products are exported to. At the same time, encouraging individuals to reduce the amount of salt used during cooking remains vital, as discretionary salt still constitutes most of the salt consumed in China.
