# Supplementary material for: Salt content of sauces in the UK and China: cross-sectional surveys
Source: BMJ Open. 2019 Sep 23;9(9):e025623. doi: 10.1136/bmjopen-2018-025623 (PMC6773338; doi:10.1136/bmjopen-2018-025623)
Supplement: Supplementary file 1 [file bmjopen-2018-025623supp001.pdf]

Supplementary table 1. Description of the sauce categories with the corresponding salt targets set by the UK Department of Health (DoH).

Sauce categories	Description	Examples	Salt targets (g/100g)						
			UK DoH's categories	2010		2012		2017	
				avg	max	avg	max	avg	max
**Salad dressings**	Mixtures based on oil and vinegar, with possible addition of seasoning.	Vinaigrette, French/ Thousand Island Dressing	14.5 Salad dressing		2.5		1.75		1.5
**Salad creams**	Based on mayonnaise, cream, or a cooked sauce containing eggs.	Salad cream, Caesar Dressing	14.3 Salad cream		1.8		1.75		1.58
**Pasta sauces**	Any sauces to be used on spaghetti or other kinds of pasta.	Bolognese / Carbonara / Tomato Pasta Sauce	15.1 All cook in and pasta sauces (except pesto and other thick sauces and pastes)	1.1		0.83*		0.75*	0.93
**Ketchups**	Sweet puree of tomatoes, onions, and green peppers.	Heinz Tomato Ketchup	14.1 Tomato ketchup		2.4		1.83		1.7
**Barbecue sauces**	Tomato paste-, mayonnaise-, or vinegar-based, usually used on barbecued food.	Jack Daniels Smokey BBQ Sauce	14.2 Brown sauce		1.5		1.5		1.2
**Vinegars**	Acetic acid-containing sour liquid made from apples, grapes, rice, etc.	Red Wine / Balsamic / Apple Cider / Rice Vinegar	N/A						
**Light soy sauces**	Thin cook-in and table sauce made by brewing ('fermented') or hydrolysis ('chemical') of soybeans. Excludes tamari and Kepjap Manis.	Lee Kum Kee Premium Light Soy Sauce	N/A						
**Dark soy sauces**	Darker, thicker, and less salty cook-in soy sauce obtained by prolonged ageing.	Amoy Dark Soy Sauce	N/A						
**Chilli sauces**	Spicy sauce containing chilli.	Maggi Extra Hot Chilli Sauce	N/A						
**Hoisin sauces**	Thick, sweet and spicy cook-in or table sauce mainly made from soybeans.	Lee Kum Kee Hoisin Sauce	15.2 Pesto and other thick sauces	3		1.5*	2	1.38*	1.63
**Bean pastes**	Savoury or spicy fermented paste made from salted soybeans.	Lee Kum Kee Black Bean Garlic Sauce	15.3 Thick pastes				5	3.25*	3.75
**Stocks (as sold)**	Dehydrated broth. To match Chinese products, this survey included cubes, granules, free powders (including pure MSG, a crystalline powder produced by bacterial fermentation) and excluded stock pots, reduction jellies, and ready-to-use products.	Knorr Vegetable Stock Pot	N/A‡						
**Mayonnaises**	Thick emulsion of raw egg yolks and oil. Excludes reduced fat/calorie versions.	Hellmann's Real Mayonnaise	14.4.1 Mayonnaise (not reduced fat/calorie)		1.5		1.25		1.25
**Low-fat mayonnaises**	Thick emulsion of raw egg yolks and oil. Only reduced fat/calorie versions.	Hellmann's Light Mayonnaise	14.4.2 Mayonnaise (reduced fat/calorie only)		2.5		1.88		1.7
**Curry pastes**	Sticky, concentrated blend of vegetables and spices; base for curry.	Mae Ploy Red Curry Paste	15.3 Thick pastes				5	3.25*	3.75
**Curry cooking sauces**	Similar to curry pastes, but prepared into a ready-to-use liquid sauce.	Uncle Ben's Medium Curry Sauce	15.1 All cook in and pasta sauces (except pesto and other thick sauces and pastes)	1.1		0.83*		0.75*	0.93
**Oyster sauces**	Viscous cook-in sauce containing oyster essence or extract.	Lee Kum Kee Premium Oyster Sauce	15.2 Pesto and other thick sauces	3		1.5*	2	1.38*	1.63
**Marinades**	Savoury sauce in which meat, fish, or vegetables are soaked to enrich flavour or tenderise.	Blue Dragon Teriyaki Marinade	N/A						

avg = average; max = maximum; MSG = monosodium glutamate
*average r: to be calculated on a sales-weighted basis.
‡The salt target set for the category '28.1 Stocks, as consumed' is not applicable here as it is meant for made-up product, as opposed to unprepared stocks, 'as sold'.

Supplementary table 2. Salt content (g/100g or ml) of sauces in China and UK, per category, 2008-18 (per year), change in median salt content of UK product over the years.

		Vinegars		Dark soy sauces		Light soy sauces			Oyster sauces		Stocks (as sold)			Marinades		Curry cooking sauces		Bean pastes		Hoisin sauces	
		CN	UK	CN	UK	CN	UK	UK†	CN	UK	CN	UK	UK†	CN	UK	CN	UK	CN	UK	CN	UK
2007-08	min				14.00		9.00	13.25											1.70		
	Q1				14.09		14.04	14.50											2.40		
	median				14.25		15.50	16.67											3.40		
	Q3				15.00		17.88	18.00											3.47		
	max				17.20		22.50	22.50											3.80		
	mean				14.91		15.89	16.60											2.96		
	sd				1.34		3.28	2.65											0.88		
	n				5.00		19.00	17.00											5.00		
2009	min																				
	Q1																				
	median																				
	Q3																				
	max																				
	mean																				
	sd																				
	n																				
2010	min																0.10				
	Q1																0.70				
	median																0.80				
	Q3																1.00				
	max																4.00				
	mean																0.91				
	sd																0.58				
	n																186.00				

		Curry pastes		Pasta sauces		Ketchups			Barbecue sauces		Chilli sauces		Salad dressings		Salad creams		Mayonnaises		Low-fat mayo.	
		CN	UK	CN	UK	CN	UK	UK†	CN	UK	CN	UK	CN	UK	CN	UK	CN	UK	CN	UK
2007-08	min						1.08	1.08				2.50		0.25		0.80		1.30		1.80
	Q1						2.02	2.32				2.86		1.30		1.80		1.30		2.25
	median						2.65	2.81				3.50		1.67		2.10		1.50		2.30
	Q3						3.08	3.10				3.59		2.30		2.65		1.50		2.50
	max						3.50	3.50				4.10		3.00		3.10		2.05		2.80
	mean						2.44	2.59				3.29		1.69		2.14		1.48		2.31
	sd						0.78	0.72				0.56		0.78		0.67		0.23		0.27
	n						14.00	12.00				7.00		13.00		15.00		10.00		11.00
2009	min				0.10															
	Q1				0.59															
	median				0.80															
	Q3				1.00															
	max				3.00															
	mean				0.83															
	sd				0.40															
	n				200.00															
2010	min		0.97				0.40	0.43		0.80										
	Q1		2.00				0.93	0.98		1.03										
	median		3.30				1.10	1.10		1.23										
	Q3		3.40				2.03	1.90		1.81										
	max		5.65				3.10	3.10		2.50										
	mean		2.84				1.47	1.49		1.44										
	sd		1.09				0.80	0.79		0.63										
	n		27.00				22.00	20.00		10.00										

CN=China; UK=United Kingdom
†excluding reduced-salt products.

Supplementary table 2. Cont.

		Vinegars		Dark soy sauces		Light soy sauces			Oyster sauces		Stocks (as sold)			Marinades		Curry cooking sauces		Bean pastes		Hoisin sauces	
		CN	UK	CN	UK	CN	UK	UK†	CN	UK	CN	UK	UK†	CN	UK	CN	UK	CN	UK	CN	UK
2011	min																				
	Q1																				
	median																				
	Q3																				
	max																				
	mean																				
	sd																				
	n																				
2012	min																				
	Q1																				
	median																				
	Q3																				
	max																				
	mean																				
	sd																				
	n																				
2013	min		0.00									14.00	14.00		0.28		0.40				
	Q1		0.00									15.00	15.00		0.73		0.69				
	median		0.00									15.00	15.00		1.13		0.73				
	Q3		0.00									15.76	15.76		1.56		0.80				
	max		0.10									16.65	16.65		2.00		0.95				
	mean		0.02									15.28	15.28		1.14		0.71				
	sd		0.04									0.99	0.99		0.62		0.16				
	n		11.00									5.00	5.00		7.00		24.00				

		Curry pastes		Pasta sauces		Ketchups			Barbecue sauces		Chilli sauces		Salad dressings		Salad creams		Mayonnaises		Low-fat mayo.	
		CN	UK	CN	UK	CN	UK	UK†	CN	UK	CN	UK	CN	UK	CN	UK	CN	UK	CN	UK
2011	min																			
	Q1																			
	median																			
	Q3																			
	max																			
	mean																			
	sd																			
	n																			
2012	min				0.10															
	Q1				0.39															
	median				0.60															
	Q3				1.10															
	max				1.82															
	mean				0.75															
	sd				0.45															
	n				30.00															
2013	min		0.97		0.20		0.20	0.20				0.60		0.00		0.80		0.48		
	Q1		1.20		0.51		0.75	0.75				0.85		0.85		1.03		0.94		
	median		2.29		0.69		0.90	0.90				2.38		1.30		1.28		1.05		
	Q3		3.00		0.80		0.90	0.90				3.80		1.52		1.60		1.30		
	max		4.20		1.70		1.33	1.33				6.43		2.20		2.70		1.80		
	mean		2.34		0.70		0.82	0.82				2.67		1.20		1.39		1.14		
	sd		1.17		0.25		0.37	0.37				2.27		0.48		0.45		0.35		
	n		14.00		66.00		6.00	6.00				7.00		27.00		18.00		16.00		

CN=China; UK=United Kingdom
†excluding reduced-salt products.

Supplementary table 2. Cont.

		Vinegars		Dark soy sauces		Light soy sauces			Oyster sauces		Stocks (as sold)			Marinades		Curry cooking sauces		Bean pastes		Hoisin sauces	
		CN	UK	CN	UK	CN	UK	UK†	CN	UK	CN	UK	UK†	CN	UK	CN	UK	CN	UK	CN	UK
2014	min		0.00									8.20	8.20				0.48				
	Q1		0.00									16.40	14.35				0.71				
	median		0.00									20.65	23.55				0.78				
	Q3		0.08									30.70	31.70				1.18				
	max		0.10									34.70	34.70				2.60				
	mean		0.03									22.13	22.50				0.99				
	sd		0.05									10.73	12.35				0.50				
	n		6.00									5.00	4.00				18.00				
2015	min	0.00	0.10	10.70		6.88			10.32		1.67	14.00	14.00		0.28		0.33	2.08	0.50	5.88	
	Q1	0.45	0.10	16.08		15.92			11.06		37.59	15.00	15.00		1.20		0.70	6.85	0.50	7.37	
	median	1.18	0.10	18.49		16.95			11.31		43.22	38.50	38.50		1.40		0.73	10.47	0.50	7.68	
	Q3	1.76	0.10	20.36		18.39			13.20		47.28	40.50	40.50		1.72		0.80	13.16	0.66	8.97	
	max	19.79	0.10	26.13		22.50			13.75		59.42	41.50	41.50		2.58		1.60	32.00	0.81	11.74	
	mean	1.41	0.10	18.46		17.21			11.94		40.58	29.90	29.90		1.42		0.74	11.49	0.60	8.37	
	sd	2.02	NA	3.30		2.53			1.24		11.43	14.10	14.10		0.62		0.16	6.60	0.18	1.79	
	n	107.00	1.00	34.00		54.00			19.00		55.00	5.00	5.00		12.00		88.00	122.00	3.00	19.00	
2016	min	0.02		13.10	13.30	12.03	14.30	14.30			12.92				0.28		0.43	3.64	0.81		
	Q1	0.80		16.95	13.78	15.75	14.38	14.38			38.87				1.36		0.69	6.64	1.09		
	median	1.32		20.13	14.25	16.80	14.45	14.45			50.00				1.90		0.73	7.75	1.38		
	Q3	1.97		24.90	14.93	18.25	14.53	14.53			52.50				2.34		0.84	10.28	1.84		
	max	8.50		24.90	15.60	21.20	14.60	14.60			57.50				5.13		1.30	20.00	2.30		
	mean	1.54		20.00	14.38	16.82	14.45	14.45			45.26				2.23		0.79	8.80	1.50		
	sd	1.70		5.12	1.16	2.58	0.21	0.21			11.36				1.46		0.20	4.03	0.75		
	n	25.00		5.00	3.00	14.00	2.00	2.00			16.00				15.00		40.00	18.00	3.00		

		Curry pastes		Pasta sauces		Ketchups			Barbecue sauces		Chilli sauces		Salad dressings		Salad creams		Mayonnaises		Low-fat mayo.	
		CN	UK	CN	UK	CN	UK	UK†	CN	UK	CN	UK	CN	UK	CN	UK	CN	UK	CN	UK
2014	min		1.30		0.30		0.30	0.30				0.04				0.70		0.90		0.79
	Q1		4.15		0.70		1.05	0.99				0.71				1.10		1.07		1.35
	median		4.65		0.80		1.45	1.52				1.20				1.30		1.18		1.60
	Q3		4.70		0.90		1.80	1.80				3.00				1.63		1.46		1.70
	max		4.80		2.00		2.10	2.10				6.70				1.70		1.78		1.98
	mean		4.02		0.83		1.37	1.37				2.01				1.32		1.26		1.53
	sd		1.36		0.30		0.50	0.51				2.04				0.30		0.28		0.28
	n		6.00		44.00		25.00	24.00				17.00				17.00		16.00		19.00
2015	min	12.52	1.30	0.68	0.20	0.15	0.30	0.30		0.61	0.07	0.00	1.85	0.20	0.50	1.00		0.85		1.30
	Q1	12.52	2.57	0.90	0.60	1.01	1.12	1.10		0.89	4.13	0.71	3.83	1.00	1.55	1.40		1.07		1.60
	median	12.52	3.10	1.12	0.74	2.53	1.60	1.60		1.10	7.50	1.40	3.93	1.30	1.91	1.56		1.18		1.66
	Q3	12.52	4.70	1.34	0.83	2.84	1.80	1.80		1.40	9.85	1.80	4.71	1.50	2.92	1.70		1.50		1.70
	max	12.52	4.80	1.55	2.30	3.45	2.21	2.21		1.50	25.00	8.40	5.44	2.29	4.13	2.20		1.80		1.80
	mean	12.52	3.41	1.12	0.78	2.03	1.43	1.44		1.09	7.61	1.87	3.95	1.30	2.21	1.56		1.27		1.62
	sd	NA	1.15	0.62	0.28	1.26	0.50	0.51		0.33	4.89	2.00	1.35	0.45	0.86	0.29		0.29		0.16
	n	1.00	16.00	2.00	133.00	10.00	22.00	21.00		9.00	68.00	17.00	5.00	32.00	25.00	20.00		28.00		19.00
2016	min		1.00	0.30	0.20	0.25	0.98	0.98			0.66	0.59	4.29	0.78	2.99	1.20	1.50	0.96		1.60
	Q1		1.33	1.17	0.60	1.41	1.22	1.22			3.62	1.25	4.29	1.02	2.99	1.37	1.50	1.16		1.60
	median		1.57	1.31	0.71	2.58	1.65	1.65			5.40	2.03	4.29	1.25	2.99	1.45	1.50	1.30		1.66
	Q3		2.32	1.39	0.81	2.73	1.73	1.73			8.75	2.77	4.29	1.40	2.99	1.52	1.50	1.45		1.69
	max		2.98	1.52	2.30	2.88	2.21	2.21			15.74	3.32	4.29	1.40	2.99	2.03	1.50	1.72		1.98
	mean		1.79	1.16	0.78	1.90	1.55	1.55			6.45	1.94	4.29	1.19	2.99	1.48	1.50	1.31		1.68
	sd		0.74	0.45	0.30	1.44	0.40	0.40			4.03	0.92	NA	0.23	0.00	0.25	0.00	0.24		0.11
	n		9.00	6.00	84.00	3.00	8.00	8.00			35.00	15.00	1.00	10.00	3.00	8.00	2.00	11.00		10.00

CN=China; UK=United Kingdom
†excluding reduced-salt products.

Supplementary table 2. Cont.

		Vinegars		Dark soy sauces		Light soy sauces			Oyster sauces		Stocks (as sold)			Marinades		Curry cooking sauces		Bean pastes		Hoisin sauces	
		CN	UK	CN	UK	CN	UK	UK†	CN	UK	CN	UK	UK†	CN	UK	CN	UK	CN	UK	CN	UK
2017	min	0.00	0.00	15.75	10.51	1.37	9.10	12.70	0.26	0.98	0.85	12.16	13.90				0.30	1.03	0.73	1.18	0.65
	Q1	1.23	0.02	16.85	13.78	15.70	11.30	13.65	1.23	1.24	45.18	15.79	16.72				0.66	10.32	0.73	4.32	1.05
	median	1.49	0.07	18.28	14.70	17.03	12.70	14.60	10.92	1.57	50.00	18.93	20.61				0.72	15.42	0.81	7.46	1.45
	Q3	1.92	0.10	20.35	15.70	17.70	14.60	15.75	11.56	3.26	52.50	37.50	40.90				0.78	21.65	0.84	9.59	3.79
	max	15.25	0.11	25.32	19.30	21.20	16.90	16.90	13.20	7.65	60.25	44.60	44.60				1.70	37.50	1.00	11.72	8.00
	mean	2.15	0.06	18.82	14.78	15.75	12.92	14.73	7.63	2.94	42.83	25.47	26.80				0.74	16.50	0.82	6.79	2.84
	sd	2.57	0.05	2.25	2.67	4.40	3.00	2.10	5.79	3.16	17.49	13.17	13.08				0.21	9.20	0.11	5.30	2.88
	n	38.00	4.00	28.00	7.00	41.00	5.00	3.00	8.00	4.00	27.00	11.00	10.00				106.00	38.00	5.00	3.00	6.00
2018	min				10.51		9.10	9.16							0.28		0.60		0.50		0.65
	Q1				14.05		12.30	12.93							1.55		0.66		0.73		1.22
	median				14.70		13.00	13.65							2.39		0.75		1.00		1.40
	Q3				15.80		14.30	15.05							7.47		0.88		1.46		2.95
	max				19.30		18.70	18.70							11.50		1.60		4.30		8.00
	mean				14.73		13.31	14.03							4.50		0.80		1.24		2.45
	sd				2.58		2.72	2.60							4.63		0.23		0.95		2.07
	n				9.00		13.00	10.00							6.00		17.00		15.00		18.00
2008-18	Fligner-Killeen test (variance)		0.4		0.5		0.3	0.3		0.9		0.1	0.06		0.09		9E-08		0.2		0.4
	Kruskall Wallis / Mood's median test		0.1		1		0.07	0.1		0.7		0.4	0.3		0.09		0.002		0.008		0.8
	Significance codes		ns		ns		ns	ns		ns		ns	ns		ns		**		**		ns
	oldest median salt value available		0.00		14.25		15.50	16.67				15.00	15.00		1.13		0.80		3.40		1.45
	newest median salt value available		0.07		14.70		13.00	13.65				18.93	20.61		2.39		0.75		1.00		1.40
	% change in median salt content		#DIV/0!		3.2%		-16.1%	-18.1%				26.2%	37.4%		111.5%		-6.3%		-70.6%		-3.1%

		Curry pastes		Pasta sauces		Ketchups			Barbecue sauces		Chilli sauces		Salad dressings		Salad creams		Mayonnaises		Low-fat mayo.	
		CN	UK	CN	UK	CN	UK	UK†	CN	UK	CN	UK	CN	UK	CN	UK	CN	UK	CN	UK
2017	min		1.02	0.90	0.20	0.19	0.20	0.32	0.00	0.71	0.08	0.55	0.25	0.20	0.21	0.90		0.75		0.79
	Q1		1.78	1.08	0.60	1.13	1.01	1.10	1.88	0.80	4.96	1.38	2.01	0.98	0.33	1.26		0.96		1.45
	median		3.40	1.37	0.70	2.30	1.43	1.48	5.15	1.20	7.86	2.47	2.85	1.20	1.90	1.40		1.08		1.60
	Q3		4.50	1.42	0.80	2.68	1.70	1.70	5.58	1.40	9.68	3.85	3.78	1.43	2.27	1.57		1.19		1.68
	max		14.20	1.87	4.00	4.72	3.09	3.09	7.37	1.52	13.00	10.80	5.80	2.29	3.52	2.20		4.10		1.80
	mean		3.57	1.32	0.74	2.12	1.36	1.42	3.87	1.13	7.30	3.00	2.94	1.19	1.62	1.45		1.18		1.48
	sd		2.72	0.32	0.31	1.34	0.66	0.64	2.59	0.36	3.41	2.37	2.28	0.40	1.09	0.31		0.55		0.31
	n		22.00	11.00	230.00	9.00	24.00	22.00	11.00	6.00	31.00	36.00	4.00	49.00	22.00	34.00		36.00		17.00
2018	min				0.50					0.70		0.49		0.98		1.20		0.78		0.95
	Q1				0.66					0.71		1.40		1.14		1.33		0.95		1.60
	median				0.78					0.88		2.55		1.29		1.45		1.03		1.60
	Q3				1.01					0.93		3.30		1.40		1.51		1.13		1.66
	max				1.30					3.40		6.30		1.60		1.63		1.16		1.69
	mean				0.84					1.32		2.59		1.27		1.42		1.02		1.56
	sd				0.28					1.17		1.54		0.19		0.15		0.13		0.23
	n				7.00					5.00		17.00		12.00		7.00		12.00		9.00
2008-18	Fligner-Killeen test (variance)		0.09		3E-13		0.2	0.4		0.9		0.09		0.0005		0.001		0.3		0.3
	Kruskall Wallis / Mood's median test		0.003		0.02		0.0005	0.0003		0.5		0.04		0.8		0.07		0.0004		0.000007
	Significance codes		**		*		***	***		ns		*		ns		ns		***		****
	oldest median salt value available		3.30		0.80		2.65	2.81		1.23		3.50		1.67		2.10		1.50		2.30
	newest median salt value available		3.40		0.78		1.43	1.48		0.88		2.55		1.29		1.45		1.03		1.60
	% change in median salt content		3.0%		-2.5%		-46.0%	-47.5%		-28.5%		-27.1%		-22.8%		-31.0%		-31.3%		-30.4%

CN=China; UK=United Kingdom
†excluding reduced-salt products.
ns P > 0.05; * P < 0.05; ** P < 0.01; *** P < 0.001; **** P <0.0001.

Supplementary figure 1. Median salt content (g/100 g or ml) of sauces in the UK, per category, 2008-2018.
The mayonnaises are categorised based on whether they are reduced in fat or calorie, not salt. Only sauce categories with five or more products per year are shown.
* P < 0.05; ** P < 0.01; *** P < 0.001; **** P <0.0001.

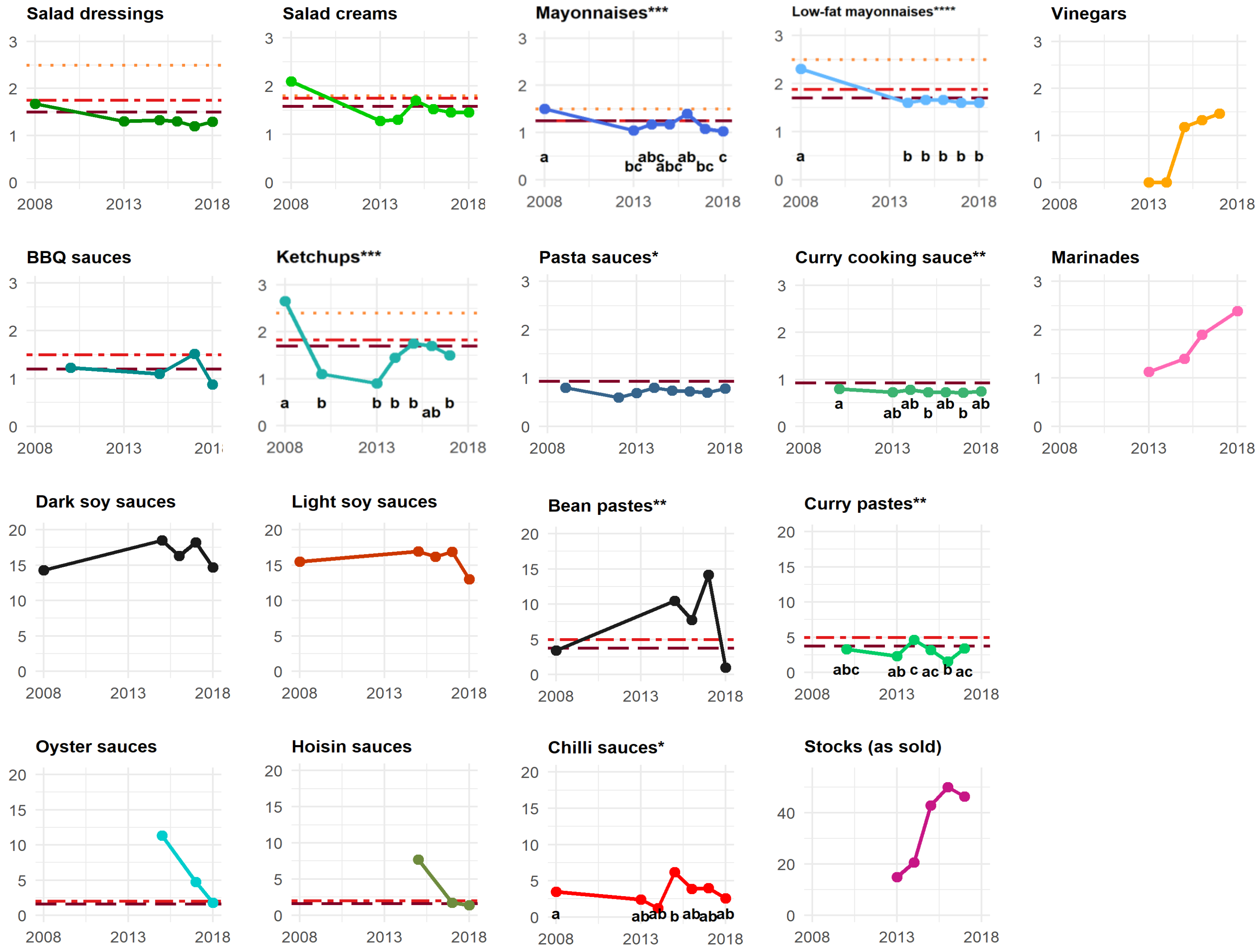

Supplementary table 3. Salt content (g/100g or ml) of sauces in China and UK, per category, 2015-17 (grouped years) and proportion (%) of products meeting the UK salt targets.

		Vinegars		Dark soy sauces		Light soy sauces			Oyster sauces		Stocks (as sold)			Marinades		Curry cooking sauces		Bean pastes		Hoisin sauces	
		CN	UK	CN	UK	CN	UK	UK†	CN	UK	CN	UK	UK†	CN	UK	CN	UK	CN	UK	CN	UK
2015-17	min	0.00	0.00	10.70	10.51	1.37	9.10	12.70	0.26	0.98	0.85	12.16	13.90		0.28		0.30	1.03	0.50	1.18	0.65
	Q1	0.80	0.03	16.45	14.01	15.75	11.65	13.90	10.86	1.24	37.80	15.79	16.72		1.29		0.68	7.18	0.73	7.37	1.05
	median	1.34	0.10	18.49	15.15	16.97	13.50	14.45	11.30	1.56	45.55	22.28	26.49		1.60		0.72	10.59	0.81	7.60	1.44
	Q3	1.88	0.10	20.37	15.65	17.70	14.52	15.17	12.91	3.26	50.00	41.00	41.25		2.08		0.80	14.15	1.00	9.08	3.79
	max	19.79	0.11	26.13	19.30	22.50	16.90	16.90	13.75	7.65	60.25	44.60	44.60		5.13		1.70	37.50	2.30	11.74	8.00
	mean	1.60	0.07	18.72	14.88	16.61	13.15	14.63	10.66	2.94	41.96	27.65	28.75		1.96		0.75	12.29	0.98	8.15	2.84
	sd	2.12	0.05	3.05	2.49	3.40	2.74	1.73	3.76	3.16	13.33	13.23	12.98		1.28		0.20	7.38	0.56	2.39	2.88
	n	170	5	67	8	109	6	4	27	4	98	15	14		22		161	178	9	22	6
	% meeting 2017 max target								11.1%	50.0%							88.8%	8.4%	100.0%	4.5%	50.0%
	% meeting 2012 max target								11.1%	75.0%								15.2%	100.0%	4.5%	66.7%
	% meeting 2010 max target																				
	Mood's median test (CN vs UK)		0.01		0.04		0.05	0.4		0.2		0.5	0.6						6.00E-09		0.05
	Significance codes		*		*		§	ns		ns		ns	ns						****		§
	Magnitude of difference		13.40		1.22		1.26	1.17		7.24		2.04	1.72						13.07		5.28

		Curry pastes		Pasta sauces		Ketchups			Barbecue sauces		Chilli sauces		Salad dressings		Salad creams		Mayonnaises		Low-fat mayonnaises		All		
		CN	UK	CN	UK	CN	UK	UK†	CN	UK	CN	UK	CN	UK	CN	UK	CN	UK	CN	UK	CN	UK	UK†
2015-17	min	12.52	1.00	0.30	0.20	0.15	0.20	0.30	0.00	0.61	0.07	0.00	0.25	0.20	0.21	0.90	1.50	0.75		0.79	0.00	0.00	0.00
	Q1	12.52	1.70	1.06	0.60	0.81	1.05	1.08	1.88	0.90	4.01	1.27	2.73	1.00	1.55	1.30	1.50	1.00		1.53	1.97	0.70	0.70
	median	12.52	2.75	1.35	0.70	2.47	1.45	1.50	5.15	1.21	7.25	2.03	3.88	1.20	1.90	1.45	1.50	1.16		1.66	8.78	0.90	0.90
	Q3	12.52	4.10	1.42	0.83	2.83	1.80	1.80	5.58	1.40	9.71	3.10	4.61	1.49	2.88	1.69	1.50	1.45		1.70	16.96	1.45	1.45
	max	12.52	14.20	1.87	4.00	4.72	3.09	3.09	7.37	1.52	25.00	10.80	5.80	2.29	4.13	2.20	1.50	4.10		1.98	60.25	44.60	44.60
	mean	12.52	3.13	1.25	0.76	2.05	1.38	1.41	3.87	1.14	7.23	2.61	3.58	1.22	2.00	1.48	1.50	1.26		1.56	12.32	1.83	1.81
	sd	NA	2.17	0.37	0.32	1.25	0.58	0.57	2.59	0.32	4.36	2.14	1.69	0.39	1.01	0.29	0.00	0.46		0.27	13.15	4.18	4.16
	n	1	41	19	325	22	45	43	11	14	134	53	10	67	50	47	2	59		31	920	918	913
	% meeting 2017 max target	0.0%	65.9%	15.8%	81.8%	31.8%	71.1%	69.8%	18.2%	50.0%			10.0%	83.6%	34.0%	68.1%	0.0%	61.0%		80.6%	13.4%	70.0%	70.8%
	% meeting 2012 max target	0.0%	97.6%			31.8%	91.1%	90.7%	27.3%	92.9%			10.0%	91.0%	36.0%	87.2%	0.0%	61.0%		96.8%	15.1%	84.7%	85.9%
	% meeting 2010 max target					45.5%	97.8%	93.3%	27.3%	92.9%			20.0%	100.0%	36.0%	89.4%	100.0%	88.1%		100.0%	44.1%	93.2%	94.4%
	Mood's median test (CN vs UK)		0.07		8.00E-08		3.00E-07	3.00E-07		0.005		0.3		2.00E-07		0.001		0.8					
	Significance codes		ns		****		****	****		**		ns		****		**		ns					
	Magnitude of difference		4.55		1.93		1.70	1.65		4.26		3.57		3.23		1.31		1.29					

CN=China; UK=United Kingdom
†excluding reduced-salt products.
ns P > 0.05; § P = 0.05; * P < 0.05; ** P < 0.01; *** P < 0.001; **** P <0.0001.
